# Biotechnological potential of *Paenarthrobacter nicotinovorans* ATCC 49919 for the microbial conversion of nicotine and tobacco extracts into 6-hydroxynicotine

**DOI:** 10.1007/s42770-026-02064-x

**Published:** 2026-08-18

**Authors:** Iustin-Tiberius Munteanu, Ana Huşleag, Robert Iustin Susanu, Andreea-Mihaela Mleşniță, Marius Mihăşan

**Affiliations:** Faculty of Biology, Alexandru Ioan Cuza University of Iaşi, Iași, Romania

**Keywords:** *Paenarthrobacter nicotinovorans*, Nicotine degradation, 6-hydroxy-L-nicotine, Bioconversion, Tobacco extract, Microbial biotechnology

## Abstract

Nicotine-rich wastes from the tobacco industry represent an environmental liability but also a potential feedstock for value-added bioconversion. In this study, the biotechnological potential of *Paenarthrobacter nicotinovorans* ATCC 49919 for selective accumulation of 6-hydroxy-(L)-nicotine (6HLN), the first intermediate of the pyridine pathway, was evaluated under flask and bioreactor conditions. Two metabolic engineering strategies were explored: transcriptional silencing of the 6-hydroxynicotine oxidase gene (*6hlnO*) using a CRISPR/dCas9 system and overexpression of the nicotine dehydrogenase large subunit (*ndhL*) to enhance entry flux into the pathway. Among the tested strains, the *ndhL*-overexpressing derivative exhibited the highest 6HLN accumulation during growth (up to 1.78 g L⁻^1^; space–time yield 104.4 mg L⁻^1^ h⁻^1^). Resting-cell bioconversion experiments identified 100 g wet cell weight L⁻^1^ as an optimal biomass loading. Chemical inhibition of 6-hydroxy-L-nicotine oxidase with 0.8 mM ZnSO_4_ further expanded the 6HLN accumulation window without substantially impairing nicotine depletion. Scale-up experiments in a stirred bioreactor demonstrated effective conversion of both pure nicotine and tobacco-derived extracts, with matrix-dependent differences in conversion kinetics. Dissolved oxygen profiles correlated reproducibly with substrate depletion and product turnover, providing a potential online process control signature. Although not achieving near-stoichiometric yields reported for knockout-based systems, the described platform offers a controllable and feedstock-flexible approach for nicotine waste valorization.

**Supplementary Information:**

The online version contains supplementary material available at 10.1007/s42770-026-02064-x.

## Introduction

(S)-Nicotine is the predominant alkaloid synthesized by tobacco plants. It is the key compound responsible for smoking addiction and one of the major toxic compounds found in by-products from the tobacco industry. Large volumes of nicotine-rich wastes are generated annually from tobacco manufacturing and cigarette production. These includes but are not limited to, tobacco field waste generated during cultivation [1], stalks, stems, midribs, and rejected dry leaves [2, 3], tobacco dust and scrap [4, 5], nicotine-rich tobacco extracts as well as tobacco wastewater [6, 7]. The recent development of electronic nicotine delivery systems has introduced a significant new category of nicotine-containing wastes that includes residual e-liquids, defective cartridges and disposable vapes [8, 9]. Some studies report that tobacco wastes with nicotine concentrations exceeding 500 ppm (dry weight) are treated as hazardous under European regulatory practice [2, 4, 10–12], but we were unable to find a primary EUR-Lex provision for this threshold.

Various reports in the literature indicate an annual production of nicotine-containing waste ranging from approximately 1.25 million to 6 million metric tons in 2010 and 2020 [13] to about 200 million metric tons in recent years [2, 14, 15]. The nicotine levels span 10^3^– 10^4^ mg/kg (d.w.) [2, 12, 16] in powdery manufacturing residues and 10⁻^1^–10^1^ mg/L in industrial or municipal wastewaters [9, 17].

A significant portion of nicotine-containing waste generated by the tobacco industry is disposed of by incineration and landfilling [2] or applied as fertilizer and pesticide [18]. This leads to air pollution and, as nicotine is water soluble, has the potential to affect the soil and groundwater. Levels ranging from ng/L to µg/L nicotine have been measured in impacted surface waters [19] and shown to promote the transfer of antibiotic resistance genes among bacteria [20]. Alternative uses of the nicotine-containing waste with a potentially lower environmental footprint exist, including production of reconstituted tobacco [13], biofuel, activated carbons/biochars and cellulose nanofibers [2]. Beside nicotine, tobacco waste also contains relevant amounts of phenols, solanesol, sterols, polysaccharides, pigments, organic acids, and minerals [5, 21], and current efforts focus on developing applications that would enable efficient utilization of this valuable bioresource [18].

Microbial transformation of nicotine from waste offers a sustainable route toward value-added intermediates that can serve as precursors for fine chemicals and pharmaceuticals such as succinic acid, methyl-γ-aminobutyric acid, 3-succinoylpyridine, and 6-hydroxy-3-succinoylpyridine [22–24]. Harnessing the capabilities of nicotine-degrading microorganisms (NDM) to convert nicotine efficiently and selectively into such products may therefore provide a dual benefit for a circular bioeconomy: waste valorization and green chemical synthesis.

Microbial nicotine catabolism proceeds via three routes – the pyridine pathway (typical of Gram-positive *Arthrobacter/Paenarthrobacter*), the pyrrolidine pathway (e.g., *Pseudomonas*) [25], and a hybrid “variant of pyridine and pyrrolidine” (VPP) pathway (e.g., *Agrobacterium tumefaciens* S33, *Shinella* sp. HZN7, *Ochrobactrum* sp. SJY1) [26].

**Fig. 1:**
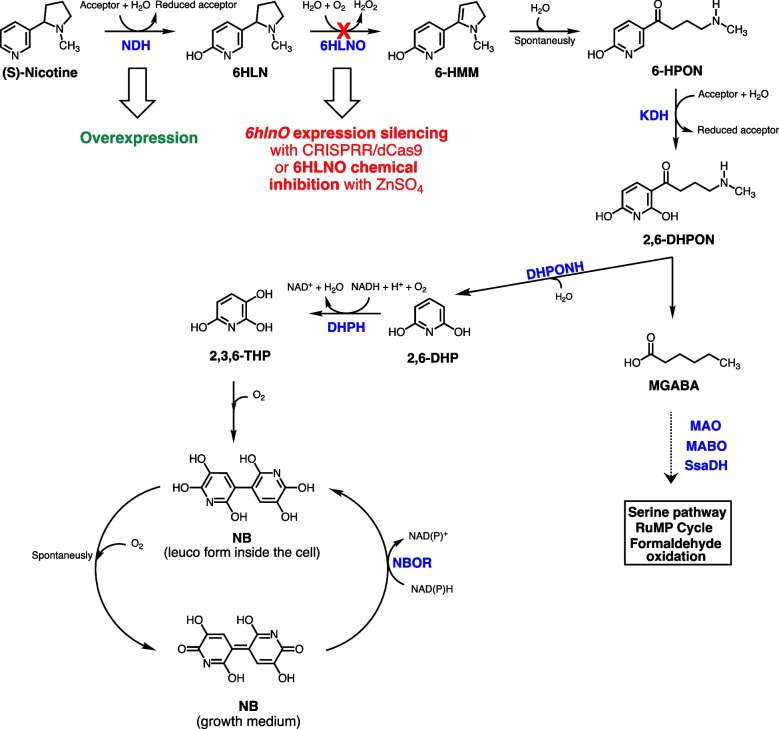
The Pyridine pathway for nicotine degradation in *P. nicotinovorans* ATCC 49919 and approaches used in this study to increase 6-hydroxy-L-nicotine yield. NDH – nicotine dehydrogenase; 6HLNO – 6-hydroxy-L-nicotine oxidase; KDH – ketone dehydrogenase; DHPONH – 2,6 dihydroxypseudooxynicotine hydrolase; DHPH – 2,6-dihydroxypyridine-3-hydroxylase; NBOR – nicotine-blue oxidoreductase; MAO – monoamine-oxidase; MABO – γ-N-methylaminobutyrate oxidase; SsaDH – succinic semialdehyde oxidase; 6HLN – 6-hydroxy-L-nicotine; 6-HMM – 6-hydroxy-methylmyosmine; 6-HPON – 6-hydroxy-pseudooxynicotine; 2,6-DHPON – 2,6-dyhydroxypseudooxynicotine; 2,6-DHP – 2,6-dihydroxypyridine; 2,3,6-THP – 2,3,6- trihydrohypyridine; NB – nicotine-blue; MGABA – γ-N-methylaminobutyrate

*Paenarthrobacter nicotinovorans* ATCC 49919 (formerly *Arthrobacter nicotinovorans* pAO1) is among the most studied nicotine-degrading microorganisms using the pyridine pathway (Fig. 1), long used as a reference system owing to its pAO1 catabolic megaplasmid and well-characterized *nic* gene cluster and enzymes [25].

This organism serves as a model for pathway operation and regulation and for the evolution and horizontal transfer of nicotine-catabolic traits among soil bacteria via plasmids [27, 28]. All steps in the nicotine catabolism, as well as the nicotine induced transcriptome and proteome of the strain has been resolved [29–31], offering a strong theoretical base for practical applications of the strain for the efficient utilization of nicotine-containing waste.

6-hydroxy-(L)-nicotine (6HLN) is the first intermediate in the pyridine pathway for nicotine catabolism *in P. nicotinovorans*. It was shown that it is a bioactive molecule with nootropic and antioxidant effects *in vivo*. In rodent models, 6HLN improved spatial memory while attenuating hippocampal oxidative stress markers (lipid peroxidation, decreased antioxidant enzymes), including in scopolamine- and chlorisondamine-induced impairment, and outperformed nicotine on several cognitive tests [32–34]. These pharmacological data, consolidated by recent reviews [35], support 6HLN as a value-added target rather than a transient intermediate and motivate bioprocesses that steer pathway flux toward its accumulation from nicotine-rich streams. Consistent with this, bioconversion routes that form 6HLN have been demonstrated in VPP-pathway bacteria (e.g., *Agrobacterium tumefaciens* S33) [36], providing proof-of-principle for targeted production from nicotine-containing substrates.

On this basis, the aim of this study was to evaluate *P. nicotinovorans* ATCC 49919 as a whole-cell biocatalyst for converting nicotine, including nicotine present in tobacco-derived extracts, into 6HLN under laboratory and bioreactor conditions. We compared the wild-type strain with recombinant derivatives designed either to increase pathway entry flux or to reduce downstream oxidation of 6HLN, in order to identify a strain background most suitable for selective intermediate accumulation. The results indicate that strain selection and process configuration both influence the extent and duration of 6HLN accumulation, and that downstream oxidation of 6HLN can be modulated.

## Methods

### Strains and growth conditions

*P. nicotinovorans* ATCC 49919 was grown on citrate medium (0.2% sodium citrate, 34 mM Na_2_HPO_4_, 22 mM KH_2_PO_4_, 0.2% (NH_4_)_2_SO_4_, yeast extract 0.5 g·L⁻^1^, pH 7.0) supplemented with 5% mineral solution and 35 μg·mL⁻^1^ kanamycin. The mineral solution consisted of 6.5 mM CaCl_2_, 20 mM ZnSO_4_, 75 mM H_3_BO_3_, 2.1 mM Na_2_MoO_4_, 0.8 mM FeSO_4_, 0.8 mM MnSO_4_, 0.4 mM CuSO_4_, 0.4 mM CoSO_4_, 15 mM KH_2_PO_4_, 8 mM MgSO_4_ and 25 mM EDTA [31]. (S)-Nicotine (Sigma-Aldrich, USA) was used across all experiments. For the engineered strains, kanamycin 140 µg·mL⁻^1^ was used for plasmid selection. All flask cultures were incubated on a rotary shaker (Model VS 60 OI, Lauda, Germany) at 28°C and 190 rpm for 24 h.

### Genetic engineering

For transcriptional gene knockdown, the pCasiART-S2(1), and pCasiART-S4(8) plasmids were assembled following the method described by Flegler and Lipski (2022). Briefly, phosphorylated, 20 bp spacers with sequences listed in Table 1 targeting the *6hlnO* gene (Gene ID: 84020294; locus_tag: JMY29_20690) were annealed and cloned into the pCasiART vector using the NEBridge® Golden Gate Assembly kit (*Bsa*I-HF v2) (New England Biolabs, USA). The ligation mixture was transformed into *E. coli* DH5α (New England Biolabs, USA), and positive colonies were selected via blue-white screening. The pART2*ndhL* strain was constructed by cloning the *ndhL* gene (Gene ID: 84020295; locus_tag: JMY29_20695) into the pART2 expression vector at the *Bam*HI and *Xba*I restriction sites as described in [37]. All sequences used in this study originate from the pAO1 catabolic megaplasmid of *P. nicotinovorans* ATCC 49919 (GeneBank accession: CP089294.1). The full coding sequences of *6hlnO* and *ndhL* are provided in the Supplementary Material*.* The strain was a kind gift from prof. Dr. Brandsch Roderich.

**Table 1:** Plasmids and primers used in this study

Plasmid	Cloned gene	Primers	Source/References
pCasiART	Constitutive vector	-	[38]
pCasiART-S2(1)	A 20 bp spacer was cloned after 5’-NGG-3’ PAM site for the *6hlnO* gene on the pAO1 plasmid (GenBank accession number: CP089294.1)	Spacer 2: Forward: GAAACGCATCACGCAGCAATGTGT Reverse: AAACACACATTGCTGCGTGATGCG	This study
pCasiART-S4(8)	A 20 bp spacer was cloned after 5’-NGG-3’ PAM site for the *6hlnO* gene on the pAO1 plasmid (GenBank accession number: CP089294.1)	Spacer 4: Forward: GAAAGAAGGCGGTGAACGTCTGGG Reverse: AAACCCCAGACGTTCACCGCCTTC	This study
pART2	Constitutive vector	-	[39]
pART2*ndhL*	pART2 vector constitutively overexpressing the large subunit of nicotine dehydrogenase NDHL (UniProtKB/TrEMBL:Q93NH5)	NDHL Forward: AATGGATAACCCACACTATTACTC NDHL Reverse: CAGGAGCAATTCTAGACTCCCTGTCC	[37]

Clone validation was performed by sequencing the plasmids on a MinION Mk1D device (Oxford Nanopore Technologies, UK). Raw reads were filtered with Chopper v0.11.0 (minimum length 1000 bp – maximum length 9500 bp) and the validation was done using both the EPI2ME wf-alignment_v.1.2.5 pipeline and Geneious Prime v2023.0.4. Electrocompetent *P. nicotinovorans* ATCC 49919 cells were prepared according Zhang et al. (2011) [46] and plasmid were electroporated using the Bio-Rad Gene Pulser (Bio-Rad, USA)

### Tobacco extract

Raw tobacco (Marlboro, Philip Morris, USA) was purchased from a local reseller. Ethanol (99% v/v), H_2_SO_4_ (98% v/v), sodium carbonate, sodium hydroxide, and sodium citrate were obtained from Chemical Company (Romania).

The procedure to obtain the alcoholic and basic extracts was based on the protocol described by Kheawfu et al. 2021 [40]. For the alcoholic extract, the dried tobacco was macerated for 48 h at room temperature (25 ± 2°C), vacuum-filtered, and concentrated using the IKA® RV 3 eco rotary evaporator (IKA-Werke GmbH, Germany) at 50°C, then evaporated to dryness and re-dissolved in distilled water. For the basic extract, 10 g of dried tobacco were suspended in 150 mL of distilled water, treated with 2 g of Na_2_CO_3_ with a 2-h incubation, autoclaved at 1.5 atm for 20 min, and subjected to sequential extraction, yielding three separate fractions with the pH of the combined extracts subsequently adjusted to 12 using NaOH.

For the acidic extraction, the procedure was based on the method described by Zheng et al. (2016) [47]. The modifications to the original protocol included a sequential extraction, yielding three separate extracts, a 2-h incubation at pH 3 prior to autoclaving at 1.5 atm for 20 min, and the use of a fixed loading ratio of 10 g raw tobacco per 150 mL of distilled water.

For the aqueous extraction, the procedure was based on the patent no CN101856143B [41]. Dried tobacco was used at a 1:10 ratio (g·mL⁻^1^ of water), boiled at 90 ± 5°C for 35 min, brought back to the initial volume with distilled water, allowed to cool to 25 ± 2°C for 1 h, and subsequently centrifuged at 4500 rpm for 30 min at 4°C.

All samples were stored at − 20°C, and the nicotine content was quantified by HPLC.

### Bioconversion assay

Wet cell weight (WCW) was determined after centrifugation at 4700 xg (RCF) for 20 min at 4°C, decanting supernatant, draining the inverted tube for 1 min, and blotting the rim to remove residual liquid. Pellets were weighed immediately on an analytical balance. Unless stated otherwise, gWCW L⁻^1^ values refer to this standardized procedure. For bioconversion at 60, 100, and 200 gWCW L⁻^1^, the reactions were carried out in flasks. Biocatalysts were introduced into distilled water containing 3 mM nicotine and 0.8 mM ZnSO_4_ and the reaction mixture was incubated at 28°C and 190 rpm. The bioconversion assay was scaled up and the substrate scoping experiments were performed in a Lambda Minifor fermenter at 10 Hz, 28°C, at a constant flow of 0.5 L/min air in a 300 mL final working volume. No pH or dissolved oxygen (partial pressure of oxygen—pO_2_) setpoints were applied during bioreactor operation. Both parameters were monitored online (in situ probes) and used only for observation/recording. The pO_2_ probe was two-point calibrated at 0% and 100% air saturation. Bioreactor runs were performed in triplicates.

### Analytical methods

Samples (0.5 mL) were collected hourly during flask screening and every 3 – 5 min during the bioconversion assays and stored at − 20°C until processed. For evaluation of bacterial growth, samples were centrifuged (20.800 xg (RCF), 3 min, 4°C), the pellet was washed thrice with distilled water, resuspended in the same volume of distilled water and monitored spectrophotometrically at 600 nm. Accumulation of the nicotine-blue pigment was monitored in the culture media supernatant at 585 nm. All measurements were performed on a DU730 spectrophotometer (Beckman Coulter, USA).

For the quantitation of nicotine and 6HLN levels, 20 μL of the growth medium supernatant were injected into a Prominence UPLC system (Shimadzu, Japan) equipped with a Chromolith® HighResolution RP-18 endcapped 150 × 4.6 mm column (Sigma-Aldrich, USA). The mobile phase was a 90:10 solution of 1 mM H_2_SO_4_: methanol at a flow rate of 1 mL/min. Separation was done at 30°C using isocratic elution [31]. Nicotine levels were measured at 250 nm, 6HLN at 290 nm. Calibration curves for (S)-nicotine (99% by GC, Sigma-Aldrich, USA) and 6HLN (98% by TLC, Santa Cruz Biotechnology, USA) were constructed by sequentially injecting 1, 5, 10, 15, 20, and 25 µL aliquots of a 0.3 mM standard solutions, with quantification based on peak area.

### Statistical analysis

Experiments were performed in triplicate and descriptive statistics was calculated using the Data Analysis—Descriptive Statistics tool in Excel (Microsoft Corporation, USA). Graphs were prepared using GraphPad Prism v10.4.2.

## Results

6HLN is the first metabolic intermediate in the pyridine pathway of *P. nicotinovorans* ATCC 49919. It is produced by nicotine dehydrogenase (NDH) and subsequently oxidized by 6-hydroxynicotine oxidase (6HLNO). 6HLN is also known to accumulate in the growth medium, probably due to differences in the catalytic efficiencies of NDH and 6HLNO [42] In order to increase the 6HLN accumulation, we first attempted to attenuate 6HLNO expression by transcriptional gene silencing using an engineered CRISPR/dCas9 system based on the pCasiART plasmid [38]. Following this strategy, two *P. nicotinovorans* recombinant strains (pCasiART-S2(1) and pCasiART-S4(8)) targeting different regions of the *6hlnO* were obtained. The second strategy employed the pART2 plasmid to constitutively overexpress NDH in *P. nicotinovorans* [39] and raise flux into the pathway.

We systematically evaluated four *P. nicotinovorans* (wild-type (WT)), pCasiART-S2(1), pCasiART-S4(8) and pART2*ndhL*) strains in flask experiments to quantify growth kinetics, nicotine depletion, and 6-hydroxy-L-nicotine formation. A lead strain was further selected for resting-cell and substrate-scope experiments to identify the most effective biocatalyst and operating conditions.

### Flask screening: growth, nicotine consumption, and 6-hydroxynicotine formation

**Fig. 2:**
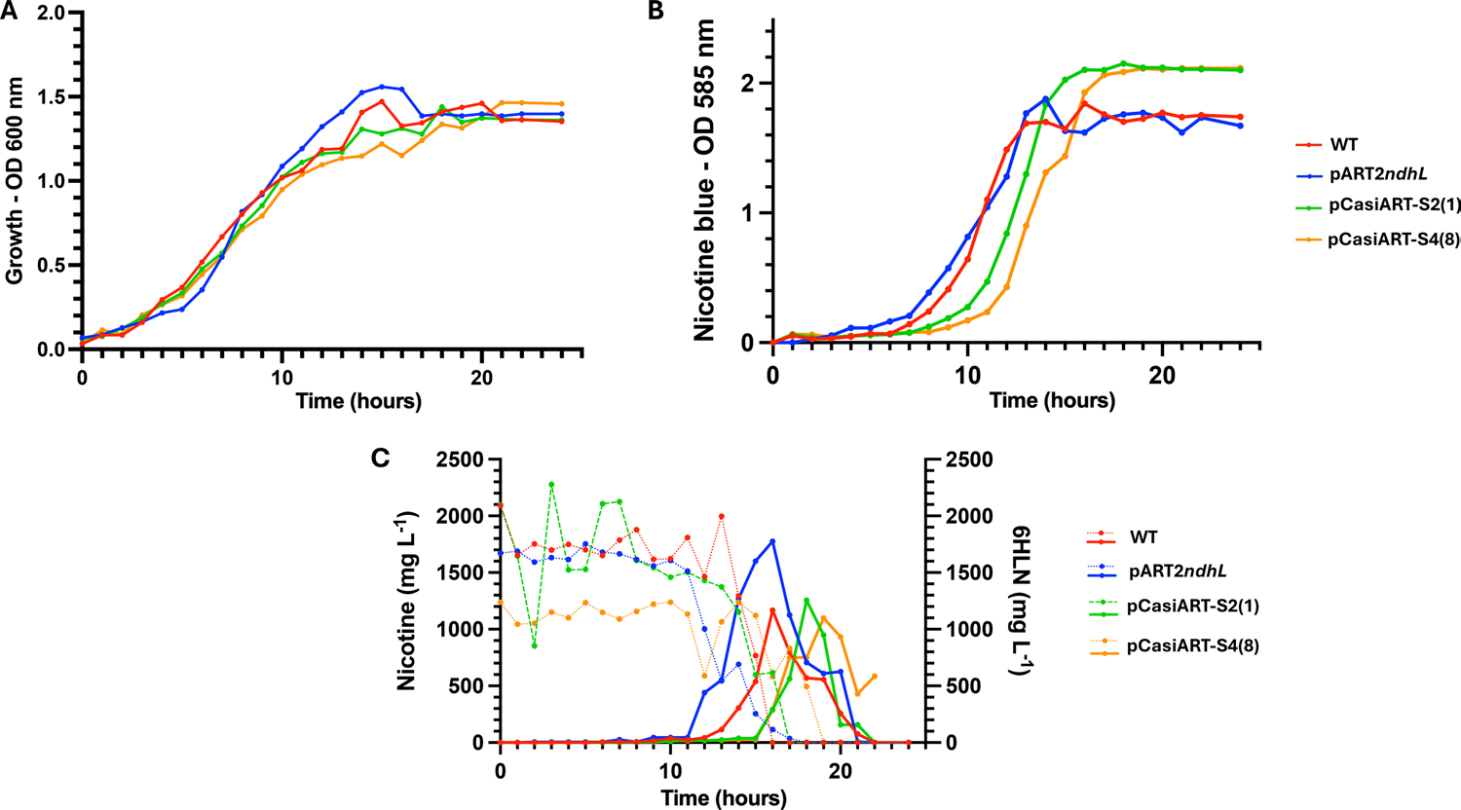
Screening for the best 6HLN producing strain. Growth kinetics (**A**), accumulation of the nicotine-blue byproduct (**B**), nicotine depletion and 6-hydroxy-L-nicotine accumulation, **C** in flask cultures of *P. nicotinovorans* WT, pCasiART-S2(1), pCasiART-S4(8) and pART2*ndhL* strains were monitored for 24 h. All experiments were performed at 11.5 mM nicotine final concentration, at 28 °C and 190 rpm

All four strains grew in citrate medium supplemented with up to 15 mM nicotine. Increasing nicotine levels prolonged the lag phase and delayed the time to maximum 6HLN accumulation (data not shown). The fastest exponential growth was observed for *P. nicotinovorans* pART2*ndhL* (μₘₐₓ = 0.41 h⁻^1^; lag ≈ 3.2 h), followed by the WT (0.29 h⁻^1^; lag ≈ 5.25 h), while pCasiART-S2(1) and pCasiART-S4(8) did not differ significantly from WT (0.28 h⁻^1^ and 0.27 h⁻^1^; lag ≈ 5.22 h and 4.25 h, respectively) (Fig. 2). Final optical densities were similar across strains (OD_600_ = 1.365 – 1.464). Nicotine half-times (t_1/2_) clustered between ~ 13 – 18 h, with pART2*ndhL* importing it the fastest (t_1/2_ = 13.7 h). The accumulation of nicotine-blue, and hence the processing of the substrate into final products takes place with relative similar speed for all strains (μₘₐₓ ranging between 0.5 to 0.66). An increased lag was nevertheless registered in the accumulation of nicotine-blue for the two pCasiART-S2(1) and pCasiART-S4(8) strains (lag ≈ 8 and 10 h compared to 6.66 h for WT and 6.11 h for pART2*ndhL*). Consistent with these observations, pART2*ndhL* exhibited the most favorable biocatalytic profile for 6HLN: max 6HLN = 1775.2 mg L⁻^1^ at 17 h (space–time yield (STY) = 104.4 mg L⁻^1^ h⁻^1^), exceeding both the WT (1167.1 mg L⁻^1^; 68.6 mg L⁻^1^ h⁻^1^) and the two pCasiART strains (1254.3 and 1099.0 mg L⁻^1^; 66.0 and 52.3 mg L⁻^1^ h⁻^1^, respectively) (Table 2). Taken together, this screening identifies pART2*ndhL* as the lead strain for downstream development, combining shorter nicotine half-times with higher productivity. In contrast, the CRISPR-based *6hlnO* silencing attempts did not increase 6HLN accumulation over WT under these conditions. Accordingly, subsequent bioconversion experiments incorporated chemical inhibition of 6HLNO to constrain downstream oxidation.

**Table 2:** Kinetic parameters of cell growth and nicotine-blue dynamics, nicotine depletion, and 6-hydroxy-L-nicotine production in flask screening

Strain	Cell growth (OD_600_)		Nicotine-blue (OD_585_)		Nicotine t_1/2_ (h)	6HLN max titer (mg L⁻^1^)	6HLN STY (mg L⁻^1^ h⁻^1^)
	lag (h)	µ_max_ (h⁻^1^)	lag (h)	µ_max_ (h⁻^1^)			
WT	5.25	0.29	6.66	0.52	15.50	1167.19	68.6
pART2*ndhL*	3.20	0.41	6.11	0.50	13.72	1775.20	104.4
pCasiART-S2(1)	5.22	0.28	8	0.56	15.20	1254.30	66
pCasiART-S4(8)	4.25	0.27	10.02	0.66	18.57	1099.01	52.3

### Resting-cell bioconversion with the lead strain

**Fig. 3:**
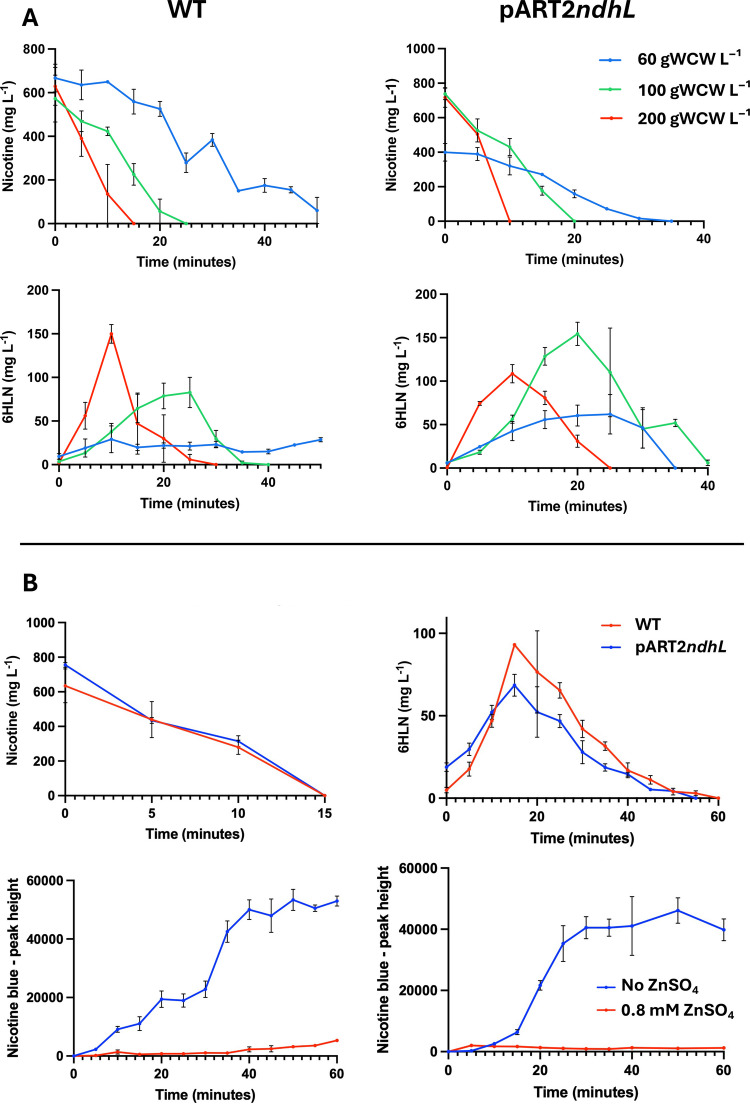
Resting-cell bioconversion with the WT and the pART2*ndhL* lead strain. **A** Influence of the of biomass loadings on the nicotine consumption (top) and 6-hydroxy-L-nicotine accumulation (bottom). **B** Influence of the 6HLNO inhibitor ZnSO_4_ on the nicotine consumption (top, left), 6-hydroxy-L-nicotine accumulation (top, right) and the accumulation of the nicotine-blue byproduct (bottom)

Based on the screening, pART2*ndhL* was selected for resting-cell bioconversion and compared with the WT strain. The resting-cell bioconversion was performed across a range of biomass loadings varying from 60 to 200 gWCW L⁻^1^ at 3 mM nicotine. For both strains, the initial nicotine consumption rate increased with biomass, but pART2*ndhL* was consistently faster (Fig. 3A). The 6HLN max titer displayed a non-monotonic dependence on biomass: pART2*ndhL* conversion peaked at 156.8 ± 35.3 mg L⁻^1^ at 100 gWCW L⁻^1^ and declined at 200 gWCW L⁻^1^ (109.2 ± 18.2 mg L⁻^1^), whereas WT increased up to 139.5 ± 4.5 mg L⁻^1^ at 200 gWCW L⁻^1^. The time required to reach peak accumulation of 6HLN ranged from 20 to 30 min at lower loadings to 10 min at higher loadings. These trends suggest that, as expected, increasing entry-point flux via NDH overexpression accelerates nicotine depletion but, at very high biomass, downstream turnover or mass-transfer limits the maximum attainable 6HLN concentration.

On this basis, 100 gWCW L⁻^1^ was selected as the working inoculum and, because genetic *6hlnO* suppression was ineffective, we next used chemical inhibition of 6HLNO to further enhance 6HLN accumulation. Addition of 0.8 mM ZnSO_4_ (Fig. 3B) increased the accumulation of 6HLN in the WT strain to 101.5 ± 0.2 mg L⁻^1^, with impact also on the initial nicotine consumption rate – nicotine t_1/2_ = 12.55 min without inhibitor, t_1/2_ = 8.35 min with inhibitor. The net effect delineates an operative condition where downstream oxidation is constrained without materially impairing substrate turnover, thereby improving the fraction of nicotine routed to 6HLN.

### Substrate scoping experiments

**Fig. 4:**
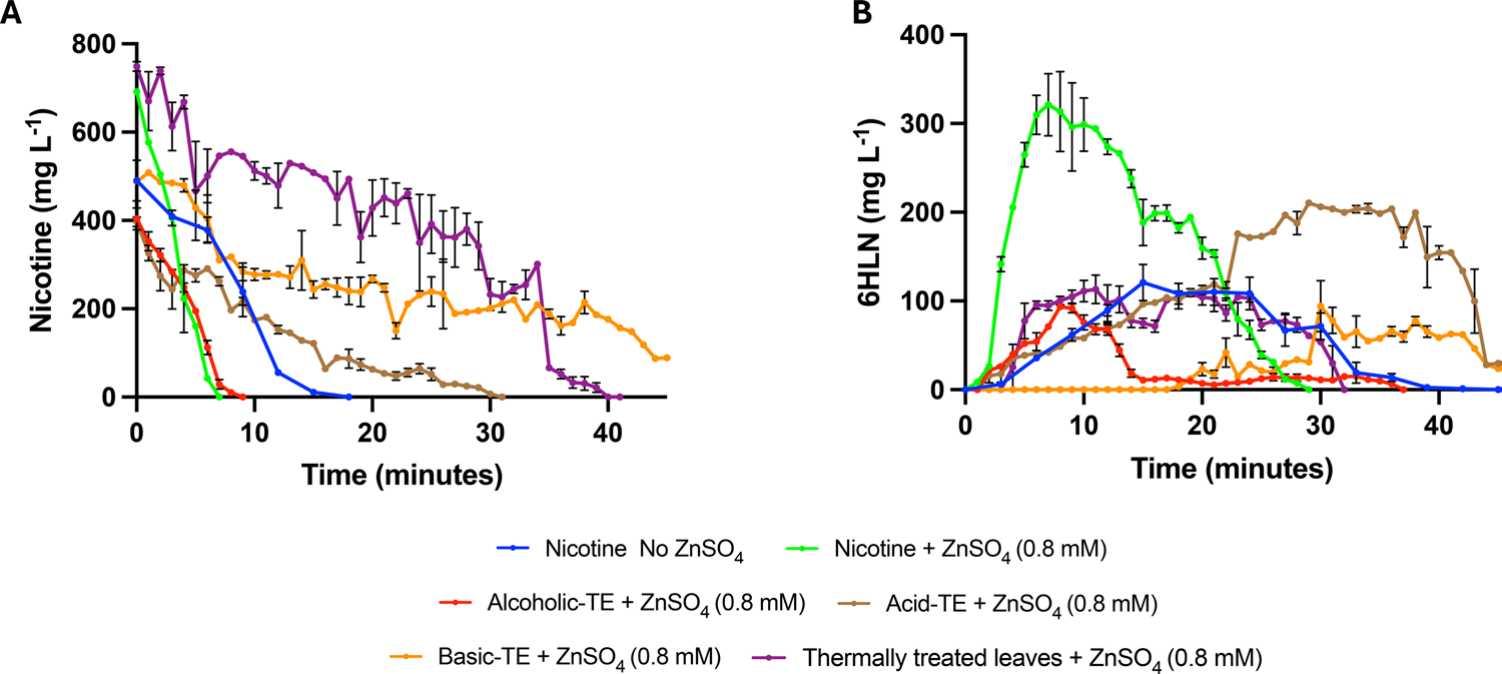
Resting-cell bioconversion of various nicotine extracts using the pART2*ndhL* lead strain (**A** – nicotine consumption; **B** – 6-hydroxy-L-nicotine accumulation)

The bioconversion with the optimized inoculum and inhibitor conditions was further scaled up and substrate scoping experiments were performed in a Lambda Minifor fermenter. As shown in Table 3, the pART2*ndhL* strain was able to convert nicotine into 6HLN from various sources, including pure nicotine, alcoholic, acidic and basic aqueous tobacco extracts as well as thermally treated leaves, albeit with different efficiency. Contaminants in the various tobacco extracts slow down both nicotine intake and degradation to different degrees, and the alcoholic extract also diminished the 6HLNO inhibitory effect of ZnSO_4_ (Fig. 4).

**Table 3:** Substrate-scope performance of two *P.nicotinovorans* strains for production of 6-hydroxy-L-nicotine in resting-cell bioconversions

Strain	WT		pART2*ndhL*					
Nicotine source*	(S)-Nicotine		(S)-Nicotine		Tobacco extract			Thermally treated leaves
					Alcoholic	Acidic	Basic	
ZnSO_4_ **	-	+	-	+	+	+	+	+
Nicotine t_1_/_2_ (min)	10.11	16.19	7.96	3.61	4.67	9.77	27.51	28.74
6HLN max titer (mg L⁻^1^)	70.74	249.84	140.45	358.51	97.05	210.49	122.63	129.06
6HLN STY (mg L⁻^1^ h⁻^1^)	283.2	535.2	561.6	2688.6	646.8	435.6	245.4	283.2

* For all conditions, substrates were dosed to the equivalent of approx. 3 mM (0.5 g/L) nicotine in the reaction mixture

** Where indicated, ZnSO_4_ was added as a 6HLNO inhibition strategy at a final concentration of 0.8 mM

**Fig. 5:**
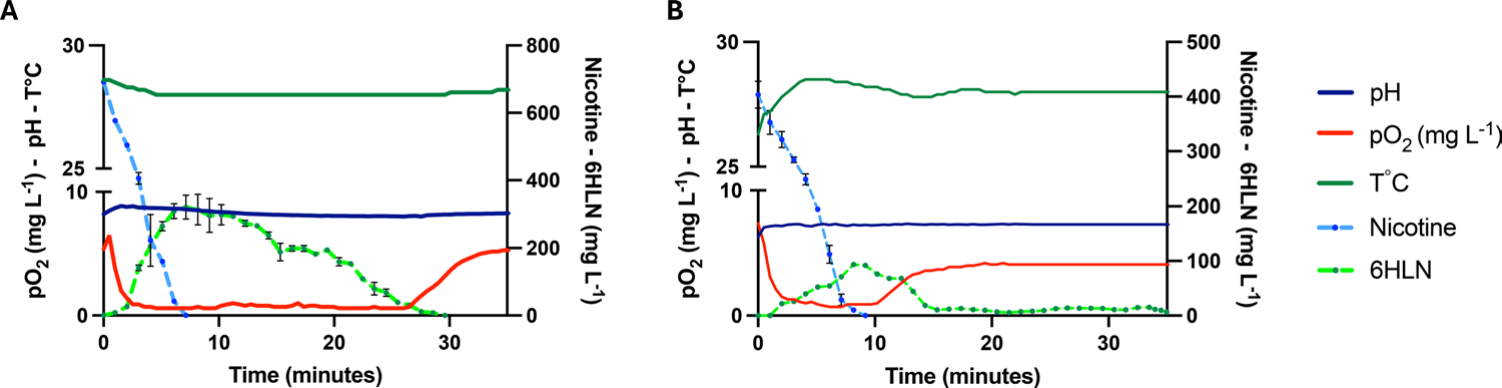
Typical dynamics of pO_2_, pH, nicotine and 6-hydroxy-L-nicotine levels during the resting-cell bioconversion of (**A**) nicotine and (**B**) the alcoholic tobacco-extract using pART2*ndhL* nicotine-induced cells

The most efficient tobacco extract is the acidic one, providing not only the highest titer but also an accumulation window of more than 20 min. One key feature of the bioconversion system reported here is that the pO_2_ in the reaction can be correlated with the nicotine and 6HLN titers. As can be observed in two examples in Fig. 5, the pO_2_ drops severely at the beginning of the bioconversion and within approx. 5 min from the minimum point, the nicotine is depleted. The pO_2_ returns to higher levels only after the 6HLN is further metabolized by the cells.

## Discussion

### How the *P. nicotinovorans* ATCC 49919 system compares with benchmark 6HLN production platforms

This work sets out to repurpose the *P. nicotinovorans* ATCC 49919 nicotine-catabolic system from production of nicotine-blue toward selective accumulation of the early intermediate 6HLN. Mechanistically, the pyridine pathway offers a clear “branch point” for such steering: nicotine dehydrogenase (NDH) introduces the 6-hydroxyl group (entry flux), whereas 6-hydroxy-L-nicotine oxidase (6HLNO) rapidly channels 6HLN onward into downstream catabolism and, ultimately, nicotine-blue formation under aerobic conditions. Consistent with this, our data show that increasing entry flux by constitutive NDH overexpression in the pART2*ndhL* strain improves 6HLN titers and STY during growth, and that constraining the 6HLNO step is critical for maintaining a practical “accumulation window” during high-rate, resting-cell conversions.

A useful comparator to our system is the engineered *Agrobacterium tumefaciens* S33 Δ*hno* system based on the developed in the hybrid/VPP nicotine pathway. Here, the deletion of the gene encoding 6-hydroxynicotine oxidase (*hno*) blocks nicotine catabolism at 6HLN and enables near-quantitative conversion: reported molar conversion reaches ~ 98%, with a specific catalytic rate up to ~ 1.01 g 6HLN h⁻^1^ g⁻^1^ dry cells under optimized whole-cell conditions; fed-batch operation converted a total of 4 g L⁻^1^ nicotine into 4.70 g L⁻^1^ 6HLN [36].

In contrast, our CRISPR-based transcriptional interference targeting *6hlnO* did not increase 6HLN accumulation beyond the WT in the flask screen. Several non-exclusive explanations are plausible. First, partial knockdown may be insufficient when pathway induction is strong; even reduced 6HLNO abundance could remain above the threshold required to prevent net 6HLN buildup. Second, targeting a gene on a catabolic megaplasmid may introduce copy-number or regulatory complexities. Variation in plasmid copy number during growth, strong nicotine-dependent induction, or transcriptional architecture around the target region could all blunt CRISPRi efficacy in ways that would be less problematic on a chromosomal, constitutively expressed target. Albeit extensive efforts to use the pCasiART as a suicidal plasmid and knockdown the *6hlnO* gene in *P. nicotinovorans* ATCC 49919, such a strain could not be obtained.

A second relevant comparison class includes VPP-pathway nicotine hydroxylases (e.g., VppA-type enzymes) that convert nicotine to 6HLN in Gram-negative nicotine degraders (e.g., *Ochrobactrum* sp. SJY1), including systems where key hydroxylase genes have been characterized for potential heterologous expression [43]. This work emphasizes the relevance of tobacco waste as feedstock for 6HLN formation, but most production systems still report their strongest performance on relatively defined substrates.

A key contribution of this work is demonstrating conversion not only from pure nicotine but also from tobacco-derived extracts (Table 3). This is important because tobacco waste streams carry phenolics, pigments, salts, and other organics that can alter membrane transport, stress response, oxygen transfer, and inhibitor availability [36].

Indeed, the *P. nicotinovorans* ATCC 49919 system described here does match the near-stoichiometric yield enabled by a clean *hno* knockout, but it demonstrates a highly responsive and process-controllable biocatalyst that can operate on chemically heterogeneous feedstocks – an important industrial constraint that is often minimized when pure nicotine is the only substrate.

### Chemical inhibition of 6HLNO—implications for process design

Evidence from a functionally analogous (S)-6-hydroxynicotine oxidase (NctB) from *Shinella* sp. [44] indicates that Zn^2^⁺ and other factors can strongly reduce oxidase activity in vitro. While the oxidase in *Shinella* sp. is not identical to the pAO1-encoded 6HLNO enzyme, it supports the usage of ZnSO_4_ as an operational lever to expand the 6HLN accumulation window in whole-cell conversions.

From a bioprocess standpoint, chemical suppression of 6HLNO is attractive because it can be tuned dynamically (dose, timing, feed strategy) and applied even in a non-engineered catalyst background. The trade-off is that inhibitor performance can be matrix-dependent, and downstream purification/regulatory acceptance will depend on the intended application of 6HLN and permissible residual metal levels.

### Bioreactor operability

The observed sharp drop in pO_2_ during early conversion and its recovery in a pattern correlated with substrate depletion/product turnover can be used as a useful online control signature. In nicotine catabolism, multiple steps deliver reducing equivalents into respiratory electron transfer, making oxygen demand a sensitive readout of pathway rate. This principle is well discussed in mechanistic studies of the hybrid/VPP nicotine pathway, where electron transfer from nicotine/intermediates to O_2_ via the electron transport chain is central to energy conservation [45]. Practically, this creates an engineering advantage: pO_2_ can be used to trigger harvest at peak 6HLN, inform feed strategies, and detect deviations when extract matrices interfere with inhibition or uptake.

## Conclusion

Even if a stable *6hlnO* knockout will improve yield as it does in *Agrobacterium tumefaciens* S33, *P. nicotinovorans* ATCC 49919 has several advantages that justify continued development. First, the pAO1 nicotine gene cluster and regulatory modules are among the best-characterized nicotine catabolic systems, enabling rational rather than purely empirical process engineering [29–31]. The system described here functions across multiple tobacco extracts and addresses the real-world constraints that industrial nicotine wastes bring [36]. Also, the reproducible pO_2_ signature offers a low-cost, scalable monitoring signal that is aligned with bioreactor control practice.

While not yet optimized to the “near-stoichiometric” benchmark of knockout-based systems, the strain and process described here provide a robust, controllable starting point for waste-to-value conversion of nicotine-containing streams into 6HLN.

## Supplementary Information

Below is the link to the electronic supplementary material.

Supplementary file1 (DOCX 17 KB)

## Data Availability

The data generated and reported in this manuscript can be made available upon reasonable request to the corresponding author.
