# Supplementary material for: Biotechnological potential of Paenarthrobacter nicotinovorans ATCC 49919 for the microbial conversion of nicotine and tobacco extracts into 6-hydroxynicotine
Source: Braz J Microbiol. 2026 Aug 18;57(1):239. doi: 10.1007/s42770-026-02064-x (PMC13486417; doi:10.1007/s42770-026-02064-x)
Supplement: Supplementary file 1 [file 42770_2026_2064_MOESM1_ESM.docx]

**Supplementary material**

Nucleotide sequences of *6hlnO* (Gene ID: 84020294; locus_tag: JMY29_20690) and *ndhL* (Gene ID: 84020295; locus_tag: JMY29_20695**)**

>NZ_CP089294.1:c122679-121402, gene =(S)-6-hydroxynicotine oxidase

Paenarthrobacter nicotinovorans strain ATCC 49919 plasmid pAO1, complete sequence

TTGTATGACGCGATTGTGGTTGGTGGGGGTTTCAGCGGGCTGAAAGCAGCTCGCGATCTTACCAACGCCG

GCAAGAAGGTACTTCTGCTTGAAGGCGGTGAACGTCTGGGAGGACGGGCGTATTCCCGGGAGTCCCGGAA

TGTACCTGGCCTGCGGGTCGAGATCGGTGGAGCATACCTTCACCGGAAGCACCACCCGCGTTTGGCAGCT

GAATTGGACCGGTATGGAATTCCCACTGCAGCTGCAAGCGAGTTCACCTCGTTTAGGCATCGCCTTGGAC

CGACCGCAGTAGACCAGGCATTTCCTATTCCTGGCTCAGAAGCAGTTGCGGTGGAAGCAGCCACCTACAC

ATTGCTGCGTGATGCGCACAGGATAGATCTGGAAAAGGGATTGGAAAACCAGGACCTTGAGGACCTGGAC

ATCCCACTGAACGAGTATGTTGACAAACTCGATTTGCCCCCTGTTTCTAGGCAGTTCTTGCTCGCTTGGG

CATGGAACATGCTTGGCCAGCCGGCTGACCAAGCATCCGCGCTTTGGATGCTGCAACTTGTTGCAGCGCA

TCACTACAGCATCCTAGGTGTTGTTCTCTCGCTGGATGAAGTTTTTTCCAATGGTTCCGCTGACCTTGTC

GACGCAATGAGCCAAGAAATTCCTGAAATCAGACTGCAGACTGTCGTGACCGGAATAGACCAGTCTGGCG

ATGTAGTGAACGTGACGGTCAAGGATGGGCATGCCTTCCAGGCTCATTCCGTGATTGTTGCTACTCCAAT

GAACACTTGGCGAAGGATCGTTTTCACCCCCGCACTTCCTGAACGGCGCAGGTCAGTAATCGAGGAAGGG

CATGGTGGTCAGGGTCTGAAAATTCTTATCCACGTGCGGGGAGCCGAGGCAGGAATTGAATGCGTCGGAG

ACGGAATATTTCCCACTCTCTACGATTATTGCGAGGTGTCGGAAAGCGAACGACTGTTGGTGGCTTTCAC

GGACTCAGGATCCTTCGATCCGACCGATATCGGCGCAGTCAAGGACGCGGTGCTTTACTACTTGCCGGAG

GTTGAAGTTCTGGGTATCGACTACCACGACTGGATCGCAGACCCGCTTTTCGAAGGGCCCTGGGTAGCAC

CGCGCGTGGGACAATTTAGTCGAGTGCATAAGGAACTTGGAGAGCCCGCTGGCCGGATTCATTTCGTAGG

TTCCGATGTTTCGTTAGAGTTCCCCGGATATATTGAAGGCGCACTGGAAACCGCTGAATGCGCGGTGAAC

GCAATTCTGCATTCGTAG

>NZ_CP089294.1:c125197-122747, gene = nicotine dehydroogenase (large subunit)/ xantine dehydrogenase family protein molybdopterin-binding subunit/ ndhL

Paenarthrobacter nicotinovorans strain ATCC 49919 plasmid pAO1, complete sequence

ATGGATAACCCACACTATTACTCTCCTCCCATAACAAGAAACGTGATCGGTAAGCGAGTTCCGCGAACTG

AGGACGCAAGACTTCTGACCGGGCGCGGGAAGTACCTGAACGACATCAACGTCGACGGCCAACTGCATGC

TTGTTTCGTGCGCAGCCCTGCTGCTCATGCTCAAATAACCGGCATTGATACGTCGGCCGCCTCAGAAATG

CCTGGCGTTCACCTTGTCTGGACATCCGCCGACATTGAGCAATACTGCGCCGGCATCGAAGCGCAGTACA

CCGCTGAGGGATGCGAAGCTATGACCATGCCGCTCTTGGCCAAGGACGTCGTACGTTACGTTGGAGAACC

GGTTGTATTGGTAGTCGCCGAGTCGCGAGCGGCTGCAGAAGATGCCTGTGATCTTGTAGGCCTTGAGCTT

GAGCACCTCGAGGTAGTGCTTGACCCGCGCAAGTCGATTCAAGGGGGCCCCGTAGCGAACGGCGAACGCC

CCGACAATGTAGGCATTCGCGGGCGGGCTTCTTTTGGGGACGTGGATGAGGCTTTTAGCAATGCTGAGCA

TGTGGTTTCCGCCCTGTATCACCCTGGTCGGGTGGCCGCCGCACCGATGGAGACAAGGGGGTGCCTAGCG

GACTACGAATGGACTGAGGACCGGCTTAAACTTTGGGTGTCGACGCAGATGCCCCATTACGTAAAAATGT

GCCTCAGCCTCTTTCTTGGTTTCGATGAGTCCCGAAGTGAGGTCATTTCTCCCGATACGGGTGGAGGCTT

TGGTCAGAAGGCGCACGTATTTCCTGAGGAAATGCTCATGCCCTTGGCCTCGAAGCACCTGAAGACGCCG

GTGAAATGGGTAGAGGATCGCCGGGAAAATCTTCTGGCGGGCTCACACGCCCACGAACAGTTCGTCACGA

TCCAATATGCGGCCAACGCTGAAGGACGAATAACCGGCGTTCGTACGCATGCCTTAGTTGATGGAGGCGC

CTACCACATGCCCCCGCAGACCATGGCTGTGGAATGCTGGGCGACCGCCGCAGTCACGCCAACTGGTGTA

TACGATATCCCGGCCGCTGAGTACGTCTACGAGGGGGCCGTTACCAACAAGTGCCCCATGGGCGCCTTCC

GTGGCGTTGGTTACACTGCGGGAACTCTTGCCAGAGAATCCCTCATGGATGATTTGGCGCGAAAGATGAA

GGTGTCTCCTTTTGAAATCAGGAGACGGAATGTTGTCAGGGAGTTCCCCTGGACAAATCCCCAGGGCGTC

GTTTATGAGGAGGGATCCTTTCTTGAAGTAATCGATGCGTTGGAAGAAATGGTCGATTACCCGGCCTTTC

TTAGGCGACAGGATGAAGCCCGCAAAGCCGGAAAGTACCTAGGGCTGGGAATCAGCGTTTTCGTCGAAAG

TAGCGGCGAGAGCACCGGCATGATGCAGGCTCACGGCGCAACGGATGTATTTCATGACACGGCTACCGTC

AAGATGGATTCAACCGGGTCGGTCACTATAACATCGGGATTGAACTCACAGGGTCAGGGCCATCAAACCA

CGCTCGCTCAGGTAGCGGCGGACGTGTTGGGTATTCCTTTTGAATCGATCAGTGTCGATGCGGGGACCTC

CACTAAGGGGGCCTATGGCAGCGGTACCATTGGAAGCCGAGCGGCTGTCATCGCGGGCGGGTGTGTCAAC

CGTGCAGCTTATGCCATTCGACAGAAACTGGTGGCCGTAGCTGCGAACATGCTCGAATCTTCTGAGGAAG

ATATCGTTTTGGAAGACGGCTTCGCGTCCGTCATAGGCGCTGCGGAATCACGAGTGAGCATCGCCGACGT

GGCCATGGCAGTCTACTGGGACTCATCCAAGTGGCCCGCCGGTTTTGAACCTGGTTTGGAGTTCACGAAG

GCGTGGGACACATCCCGGCCGATGTTTTCGAACGGCGGTCATGCCCTTATTGTTGAACTGGATCCTGTCA

CTGGATTTGTGAAAGTCGAAAAGGTTTATTCTGTAGAGGACTGCGGCGTTATTATCAACCCCACAATTGT

TGAAGGTCAAATTCGAGGTGGGGTCGTACAAGGAATTGGTATGGGCCTCTTCGAACAGCTGGCTTATGAC

AACGCAGGAAATTTGTCTACGACATCTTTCCTGGATTACCAGACGCCGACTATGGATGTTTCTCCGCCAT

TTGAAATCAGGCACATTGAAACACCGTCCGTGATGACAGCATCTGGGGTGAAGGGCATGGGGGAGTCGGG

TCTGATTTCGGCGCCGGCTGCGGTGCTGAATGCCGTAAATGATGCGCTATCTCCGTTTGGTTCTGTCCTC

TATGAACTCCCTGCCACTCCGGAGAAGGTTGTTCGTGCAACGAAAGGCATCATTGACCTGCAGGGCCCCC

AGGACTGGTCAGAGCTATGGGAACGGGCAGGGGTACCCGCGCTTGATCGCGGCCCTATGGACAGGGAGTA

A
